# AmrZ and FleQ Co-regulate Cellulose Production in *Pseudomonas syringae* pv. Tomato DC3000

**DOI:** 10.3389/fmicb.2019.00746

**Published:** 2019-04-17

**Authors:** Daniel Pérez-Mendoza, Antonia Felipe, María Dolores Ferreiro, Juan Sanjuán, María Trinidad Gallegos

**Affiliations:** Department of Soil Microbiology and Symbiotic Systems, Estación Experimental del Zaidín (EEZ-CSIC), Granada, Spain

**Keywords:** FleQ, AmrZ, cellulose, c-di-GMP, transcriptional regulation, *Pseudomonas syringae*

## Abstract

*Pseudomonas syringae* pv. tomato DC3000 carries the *wssABCDEFGHI* operon for the synthesis of acetylated cellulose, whose production is stimulated by increasing the intracellular levels of the second messenger c-di-GMP. This enhances air-liquid biofilm formation and generates a wrinkly colony morphotype in solid media. In the present study we show that cellulose production is a complex process regulated at multiple levels and involving different players in this bacterium. Using different *in vitro* approaches, including *E*lectrophoretic *M*obility *S*hift *A*ssay (EMSA) and footprint analysis, we demonstrated the interrelated role of two transcriptional regulators, AmrZ and FleQ, over cellulose production in Pto DC3000 and the influence of c-di-GMP in this process. Under physiological c-di-GMP levels, both regulators bind directly to adjacent regions at the *wss* promoter inhibiting its expression. However, just FleQ responds to c-di-GMP releasing from its *wss* operator site and converting from a repressor to an activator of cellulose production. The additive effect of the double *amrZ/fleQ* mutation on the expression of *wss*, together with the fact that they are not cross-regulated at the transcriptional level, suggest that FleQ and AmrZ behave as independent regulators, unlike what has been described in other *Pseudomonas* species. Furthermore, this dual co-regulation exerted by AmrZ and FleQ is not limited to cellulose production, but also affects other important phenotypes in Pto DC3000, such as motility and virulence.

## Introduction

The bis-(3′,5′)-cyclic diguanosine monophosphate (c-di-GMP, cyclic diguanylate, cdG) was discovered thirty years ago as an allosteric activator of bacterial cellulose synthase (Ross et al., 1987; Römling and Galperin, 2017), and is currently considered an universal bacterial second messenger (Römling et al., 2013). This cyclic dinucleotide influences diverse cellular processes, including cell to cell signaling, progression of cell cycle and virulence, but it is best known for regulating the transition from a planktonic and often motile lifestyle to a biofilm and sessile mode of growth (Hengge, 2009). The biofilm is a dynamic tridimensional structure where bacteria live in a self-produced matrix of lipids, nucleic acids, polysaccharides and proteins, whose structural and functional properties are essential for resource capture, enhanced survival and social cooperation (Flemming et al., 2016). Exopolysaccharides (EPS) are a major fraction of the biofilm matrix that provide a survival advantage by protecting bacterial cells against biotic and abiotic stresses, and are also involved in cell to cell interactions and surface adhesion. They are synthesized by different pathways strictly controlled by environmental cues and often sharing common regulatory schemes (Schmid et al., 2015). Currently, roughly a dozen bacterial EPS (i.e., cellulose, alginate, Pel, curdlan, etc.) are known to be controlled by c-di-GMP (Mann and Wozniak, 2012; Pérez-Mendoza and Sanjuán, 2016). Most of them are cryptic or yields are very low under laboratory conditions; nonetheless, the artificial rising of bacterial c-di-GMP levels by controlling the expression of diguanylate cyclases has facilitated their discovery (Pérez-Mendoza et al., 2011, 2014, 2015). This is because the majority of c-di-GMP regulated EPS biosynthetic machineries are multienzymatic complexes that exhibit inactive conformations. They turn on at particular and often unknown physiological or environmental conditions by c-di-GMP allosteric regulation of the synthase or other accessory proteins of the biosynthetic complex (Whitney and Howell, 2013). In addition to the post-translational regulation, c-di-GMP also modulates the transcription of many EPS biosynthetic operons through the action of different transcriptional regulators, which, upon binding c-di-GMP, change their binding affinity to EPS promoters, releasing their repression and/or activating their transcription (Hickman and Harwood, 2008; Fazli et al., 2011; Shikuma et al., 2012). Thus, artificially elevating the bacterial c-di-GMP content leads to the activation of the biosynthesis complexes and massive production of one or more EPS in multiple bacteria (Liang, 2015).

*Pseudomonas syringae* pv. tomato (Pto) DC3000 is a tractable model organism to study plant-pathogenic bacteria interactions (Preston, 2000; Buell et al., 2003; Xin and He, 2013). In nature, this strain is responsible for the bacterial speck disease on tomato (*Solanum lycopersicum*), but it also infects other plants like *Arabidopsis thaliana* and various *Brassica* species (Whalen et al., 1991; Zhao et al., 2000). Pto DC3000 is usually present on leaf surfaces, in soil, seeds and rotting plant material, but only when it enters into the leaf tissues through stomata or wounds and proliferates in the apoplast is able to cause chlorotic and necrotic lesions in susceptible plants or programmed cell death in incompatible interactions (Hirano and Upper, 2000; Xin and He, 2013). The virulence of Pto depends on a type III secretion system (T3SS), a needle-like appendage that delivers virulence effector proteins into the host cells (Collmer et al., 2000; Galan and Wolf-Watz, 2006). Pto DC3000 also produces the phytotoxin coronatine, which is a chlorosis-inducing polyketide that controls stomata opening upon bacterial infection and contributes to lesion development and bacterial proliferation and spreading in the host tissue (Bender et al., 1999; Gimenez-Ibanez et al., 2017).

Regarding EPSs, Pto DC3000 is able to synthesize alginate, levan, Psl, and acetylated cellulose. Cellulose is a linear homopolymer of D-glucose linked by β (1–4) bonds. Bacterial cellulose was first isolated in 1886 from the pellicle formed by an acetic acid bacterium now called *Komagataeibacter (Acetobacter) xylinus* (Brown, 1886). Subsequently, experimental reports of bacteria producing cellulose as well as the annotation of putative biosynthesis operons in bacterial genomes increased, suggesting that a variety of Gram-negative bacteria may be able to produce this EPS (Römling and Galperin, 2015). Cellulose is synthesized by cellulose synthases (BCS, EC 2.4.1.12), a membrane-anchored protein complex that transfers glucosyl residues from UDP-glucose to the incipient β-D-1,4-glucan chain. The first identified genes responsible for cellulose biosynthesis in bacteria were the *K. xylinus bcsABCD* operon. BcsA and BcsB are essential for the BCS activity *in vitro* whereas all four proteins are required for maximal cellulose production *in vivo*. In fact, BcsC and BcsD are involved in the export and packing process of the glucan molecules at the cell surface (Saxena et al., 1990; Wong et al., 1990; Omadjela et al., 2013). Pto DC3000 likely produces an acetylated form of cellulose thanks to the enzymes encoded in the *wssABCDEFGHI* operon (Pérez-Mendoza et al., 2014; Prada-Ramírez et al., 2016). The BCS (WssB/BcsA and WssC/BcsB), secretion of cellulose (WssA/BcsQ, WssD/BcsZ, and WssE/BcsC) and others similar to those involved in alginate acetylation (WssG/AlgF, WssH/AlgE, and WssI/AlgJ).

We previously reported that the artificial rising of intracellular c-di-GMP levels in Pto DC3000 enhanced the production of cellulose, alginate and A-L biofilm, and generated a colony morphotype similar to the rdar or WS (Rainey and Travisano, 1998; Spiers et al., 2002; Römling, 2005; Pérez-Mendoza et al., 2014). We also proved that the transcriptional regulator AmrZ (alginate and motility regulator Z), originally described as the activator of alginate production (Baynham et al., 1999), transcriptionally represses the *wss* operon. AmrZ, which belongs to the Arc family of proteins with ribbon-helix-helix (RHH) DNA binding domains, is present in all the *Pseudomonas* genomes sequenced so far and is considered a key global regulator involved in environmental sensing and adaption (Jones et al., 2014; Martínez-Granero et al., 2014). In fact, AmrZ acts as a bifunctional regulator in Pto DC3000 because it represses cellulose biosynthetic genes and numerous proteins engaged in the c-di-GMP metabolism, and activates alginate production, virulence and motility (Prada-Ramírez et al., 2016).

FleQ belongs, together with NtrC or NifA, to the family of bacterial enhancer binding proteins (bEBPs) and possesses the three characteristic domains of this family: an N-terminal REC domain, a central domain AAA+ with ATPase activity which also interacts with σ^54^, and a C-terminal Helix-Turn-Helix DNA binding domain (Arora et al., 1997; Baraquet and Harwood, 2013; Srivastava et al., 2013; Matsuyama et al., 2016). As bEBP, FleQ activates the RNA polymerase with the RpoN factor (σ^54^) thanks to its ATPase activity, but it is also capable to respond to variations in the c-di-GMP intracellular levels by modifying its activity (Arora et al., 1997; Hickman and Harwood, 2008; Su et al., 2015). FleQ is the master regulator of flagellar biogenesis in *Pseudomonas*, where it also controls surface attachment and biofilm formation in response to c-di-GMP, therefore mediating the transition between planktonic and biofilm lifestyles (Baraquet et al., 2012; Baraquet and Harwood, 2016; Jiménez-Fernández et al., 2016; Matsuyama et al., 2016; Molina-Henares et al., 2016). In Pto DC3000 a *fleQ* mutant exhibits the following phenotypes: remains immobile in swimming plates but exhibits an altered surface spreading on semisolid agar. It generates no flagella, but overproduces the biosurfactant syringafactin (Vargas et al., 2013; Nogales et al., 2015). In *Pseudomonas aeruginosa* it regulates the expression of the operons *psl* and *pel* for the production of two distinct EPS (Hickman and Harwood, 2008). Specifically, FleQ has a double function in the regulation of *pel*, since two FleQ binding motifs (box 1 and box 2) have been identified in the promoter region and it has been observed that FleQ modifies *pel* expression, repressing it from box 2 and activating from box 1. The binding of c-di-GMP, therefore, transforms FleQ from repressor to activator in a process independent of its ATPase activity (Baraquet et al., 2012). In *Pseudomonas fluorescens* Pf0-1, FleQ regulates the expression of various regulators, enzymes, putative lipoproteins and hypothetical proteins involved in motility and biofilm formation (Mastropaolo et al., 2012). In *Pseudomonas putida* KT2440 and *Pseudomonas fluorescens* SBW25, FleQ activates the transcription of the *bcs* and *wss* operons, respectively, for the synthesis and secretion of cellulose into the extracellular space in response to high concentrations of intracellular c-di-GMP (Giddens et al., 2007; Spiers et al., 2013; Jiménez-Fernández et al., 2016; Xiao et al., 2016; Nie et al., 2017).

In the present study we evidence the interrelated role of two transcriptional regulators, AmrZ and FleQ, over cellulose production in *Pseudomonas syringae* pv. tomato DC3000 and the influence of the second messenger c-di-GMP in this process. This complex regulation, at multiple levels and involving different players, likely provides a rapid and accurate control for the production of this important EPS. Furthermore, this dual co-regulation exerted by AmrZ and FleQ is not limited to cellulose production, but also affects other important phenotypes in Pto DC3000, such as motility and virulence, being very different from that described in other bacteria.

## Results

### Cyclic-di-GMP Stimulates Transcription of the *wss* Operon

**FIGURE 1:**
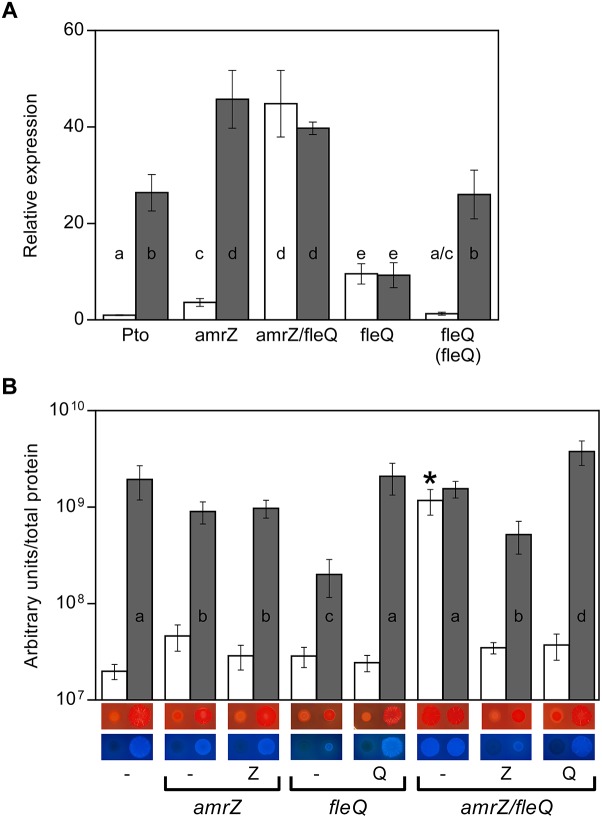
Cellulose production in Pto DC3000. **(A)** Effect of c-di-GMP on the expression of the *wss* operon in different genetic backgrounds. Total RNAs were obtained from bacteria grown in MMR at 20°C for 24 h. Results show qRT-PCR of *wssB* in the wild type strain (Pto) and mutants *amrZ*, *amrZ/fleQ* and *fleQ*, the latter also complemented, with pJB3Tc19 (in the absence of *pleD^∗^*, white bars) or with pJB3pleD^∗^ (in the presence of *pleD*^∗^, gray bars). Expression values were normalized with the housekeeping gene *gyrA* and referred to the wild type condition in the absence of *pleD*^∗^. The graph shows the average mRNA levels and error bars correspond to the standard deviation of three biological replicates; a–e denote ANOVA categories with significant differences (*P* < 0.01). **(B)** Cellulose production and colony morphology. Pto and the *amrZ*, *fleQ* and *amrZ/fleQ* mutants were grown in MMR with CF (100 μg/ml) for 24 h at 20°C, and the fluorescence emission of the cell attached CF in liquid cultures was measured. The graph shows the average amount of cellulose produced by the indicated strains in the absence (white bars) and in the presence of *pleD*^∗^ (gray bars) as fluorescence (in arbitrary units) referred to total cell protein. Error bars correspond to the standard deviation of three biological replicates and a-d denote ANOVA categories with significant differences (*P* < 0.01). Representative colony morphology of the different strains grown in agar plates supplemented with Congo Red and Calcofluor is included below. 5 μl of bacterial suspensions at OD_660_ = 1.0 were placed on the surface of MMR plates with CR (50 μg/ml, top) or CF (100 μg/ml, bottom) and pictured after incubation at 20°C for 3 days.

We previously showed that the regulation of cellulose production in Pto DC3000 occurs at various levels. The expression of the *wss* operon is negatively regulated at the transcriptional level by AmrZ, which also controls different c-di-GMP metabolizing proteins that may modulate the cellulose synthase activity at the post-translational level (Pérez-Mendoza et al., 2014; Prada-Ramírez et al., 2016). AmrZ transcriptionally represses the *wss* operon by directly binding to a specific sequence that overlaps with the -10 region of its promoter. Accordingly, an *amrZ* mutant synthesized more cellulose than the wild type and exhibited redder colonies on Congo Red (CR) plates and more fluorescent colonies under UV light on plates supplemented with Calcofluor (CF) (Prada-Ramírez et al., 2016). We also showed that the artificial increase of Pto DC3000 c-di-GMP levels generated by the overexpression of the diguanylate cyclase *pleD*^∗^ (Paul et al., 2004) stimulates cellulose production, most likely activating the cellulose synthase complex through the WssB PilZ domain (Pérez-Mendoza et al., 2014). Interestingly, we observed that *wssB* mRNA levels in the wild type strain increased about 25-fold in the presence *pleD*^∗^ (Figure 1A), indicating a dual regulation role of this second messenger that also induces the transcription of this operon from the same start site (Supplementary Figure S1). Furthermore, c-di-GMP increased *wss* transcript levels 13-fold in the *amrZ* mutant (Figure 1A). Therefore, the transcriptional activation of the *wss* operon by high c-di-GMP levels was not mediated by AmrZ, suggesting that another regulator was involved in this process. Taking into account the high homology and regulatory action of FleQ on the production of various EPSs in response to c-di-GMP in other *Pseudomonas* (Giddens et al., 2007; Hickman and Harwood, 2008; Baraquet et al., 2012; Baraquet and Harwood, 2013, 2016; Spiers et al., 2013; Jiménez-Fernández et al., 2016; Xiao et al., 2016; Nie et al., 2017), we set out to study the role of FleQ in the regulation of the cellulose synthesis operon in Pto DC3000.

### Role of FleQ and c-di-GMP in Cellulose Production

Colony morphology of the DC3000 *fleQ* mutant was studied on plates supplemented with CF and CR, both in the absence and in the presence of *pleD*^∗^ (Figure 1B). At physiological c-di-GMP levels (in the absence of *pleD*^∗^), *fleQ* produces colonies similar to those observed in the wild type strain: faintly red on plates supplemented with CR and non-fluorescent under UV light on CF plates. However, at high levels of c-di-GMP (in the presence of *pleD*^∗^) the mutant forms smooth red colonies on CR, instead of the rosettes observed with the wild type or the complemented mutant. Likewise, at high c-di-GMP levels, *fleQ* colonies were less fluorescent on CF compared to those of the wild type or the complemented strain (Figure 1B). These results were confirmed by quantifying cellulose production in liquid cultures, and observing no significant differences among the wild type, *fleQ* and the complemented mutant at physiological levels of c-di-GMP (Figure 1B). However, in the presence of *pleD*^∗^, the *fleQ* mutant increased cellulose production 7-fold relative to the strain with the empty plasmid (pJB3Tc19), but these levels were 10-fold lower than those measured with the wild type or the complemented mutant overexpressing *pleD*^∗^ (Figure 1B). These results suggest that FleQ has a dual role (positive and negative) in the regulation of cellulose synthesis in Pto DC3000 in a c-di-GMP-dependent manner and, at least in the presence of AmrZ (see below) and under high c-di-GMP, it is necessary for the maximum observed cellulose production.

**FIGURE 2:**
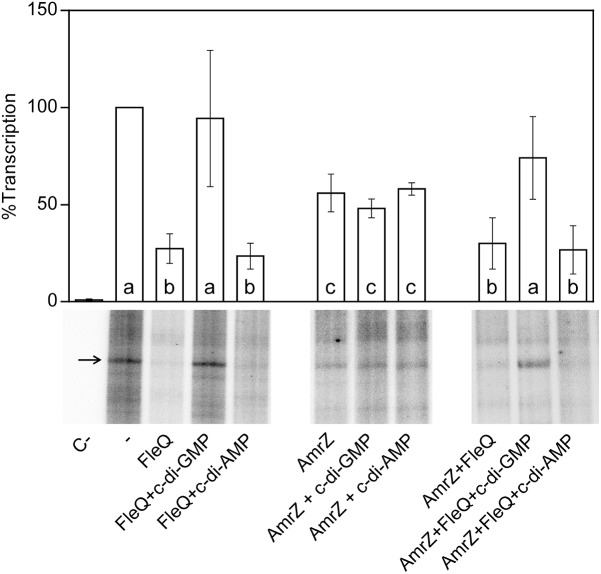
*In vitro* transcription. Multiple round transcription assays were carried out as described in Materials and Methods. The assays were performed in the absence (-) or in the presence of 0.5 μM FleQ. When indicated, c-di-GMP or c-di-AMP (0.25 mM) or AmrZ (50 nM) were also added to the reaction. C- indicates a reaction without template DNA. The 232 nucleotide mRNA synthesized from PwssA is indicated by arrowheads. The graph shows the average amount of the mRNA produced as percentage of the condition without any protein (-). Error bars correspond to the standard deviation of six different transcription assays and a–c denote ANOVA categories with significant differences (*P* < 0.01).

To test whether FleQ was regulating the transcription of the biosynthetic cellulose operon, *wssB* gene expression was measured by RT-qPCR in Pto DC3000, *fleQ* and the complemented mutant, both in the absence and in the presence of *pleD*^∗^ (Figure 1A). As it is mentioned above, *wssB* mRNA levels were low in the absence of *pleD*^∗^ but increased about 26-fold in its presence. Differently, expression of *wssB* increased about 10-fold in the *fleQ* mutant with respect to the wild type, in the absence of *pleD*^∗^ and remained the same in its presence (Figure 1A). Thus, the positive effect of c-di-GMP over *wssB* mRNA levels is dependent on the presence of FleQ. Complementation with the wild type allele almost completely restored the wild-type phenotype. Therefore, FleQ seems to repress *wss* transcription under low c-di-GMP and activate it under high c-di-GMP levels. To confirm this role of FleQ, we performed *in vitro* transcription assays both in the absence and in the presence of c-di-GMP. We observed that FleQ significantly repressed (4-fold) the expression of the *wss* operon in the absence of c-di-GMP. However, in the presence of c-di-GMP transcription was almost totally de-repressed, whereas c-di-AMP had no effect (Figure 2).

It has been reported that FleQ is able to specifically bind c-di-GMP in different Pseudomonads (Hickman and Harwood, 2008; Baraquet et al., 2012; Baraquet and Harwood, 2013; Su et al., 2015; Matsuyama et al., 2016). To corroborate that this is also the case for the Pto DC3000 FleQ ortholog, we used fluorescence-based thermal shift assays (FTSA) in the presence of different nucleotides (ATP, GTP, c-di-AMP and c-di-GMP; Supplementary Figure S2). The FTSA of ligand-free FleQ resulted in a Tm of 37.4°C. We observed that only c-di-GMP was able to significantly stabilize the protein during its thermal unfolding, increasing its ΔTm 8°C compared to FleQ on its own (Supplementary Figure S2). ATP and GTP also increased FleQ Tm but only 2.3 and 2.8°C, respectively, and c-di-AMP was not able to increase the ΔTm (Supplementary Figure S2). These results strongly suggest that Pto DC3000 FleQ binds c-di-GMP but not c-di-AMP.

### FleQ Directly Binds to the Cellulose Operon Promoter Region and c-di-GMP Influences Its Binding

**FIGURE 3:**
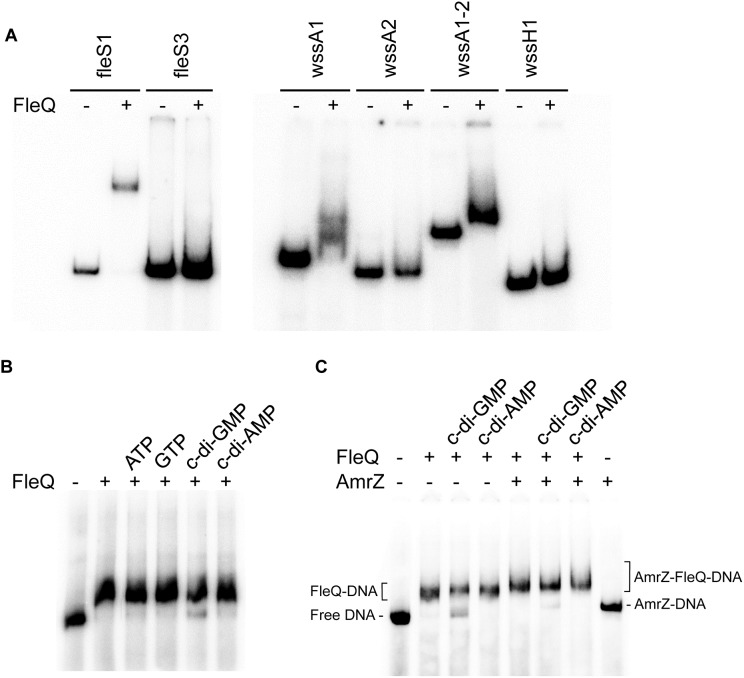
*In vitro* binding of FleQ to the *wss* promoter region. **(A)** EMSA with different fragments of the *fleSR* and *wss* operon. Binding reactions were carried out as described in Experimental Procedures. The indicated fragments were incubated without protein and with 1 μM of native FleQ. In presence of FleQ, shifted fleS3, wssA2 and wssH fragments were not observed. **(B)** Binding of FleQ to the *wss* promoter in the presence of different nucleotides. Binding reactions were carried out in the absence (-) and in the presence of 1 μM FleQ and 0.5 mM of ATP, GTP, c-di-GMP or c-di-AMP. **(C)** Binding of FleQ to the *wss* promoter in the presence of AmrZ. Binding reactions were carried out in the absence (-) and in the presence of 1 μM FleQ, 50 nM AmrZ and 0.5 mM of ATP, GTP, c-di-GMP or c-di-AMP (0.5 mM). Putative shifted FleQ-DNA, AmrZ-DNA and FleQ-AmrZ-DNA complexes are indicated.

To check whether FleQ binds directly to the *wssABCDEFGHI* operon controlling its expression, gel electrophoretic mobility shift assays (EMSA) with the purified protein and different fragments of the *wss* operon were carried out. Also, a fragment of the Pto DC3000 *fleSR* promoter was used as a positive control, similar to others of *P. aeruginosa* with which FleQ binding had been previously demonstrated (Jyot et al., 2002; Hickman and Harwood, 2008). Initially, optimal conditions were established for the binding of the native protein from Pto DC3000 to the DNA *in vitro*: 0.5–1 μM protein, 1 nM DNA and incubation on ice for 30 min. In the gel shift assays FleQ binding was only detected to the DNA fragments containing the *fleSR* and *wssA* promoters (specifically to the fleS1, wssA1, and wssA1-2 fragments), but not to other fragments of the *wss* operon (Figure 3A). We thus proceeded with further studies using only the wssA1-2 fragment since it was the best probe to assay FleQ binding to the *wss* operon under the conditions tested. It should be also noted that the addition to the reaction of the same unlabelled competitor DNA (cold DNA), but not an excess of non-specific DNA, diminished the formation of this complex, confirming that the binding observed is specific (Supplementary Figure S3). Additionally, we observed that the presence of c-di-GMP, but not c-di-AMP, ATP, or GTP, partially disrupted the FleQ-DNA complex (Figure 3B).

**FIGURE 4:**
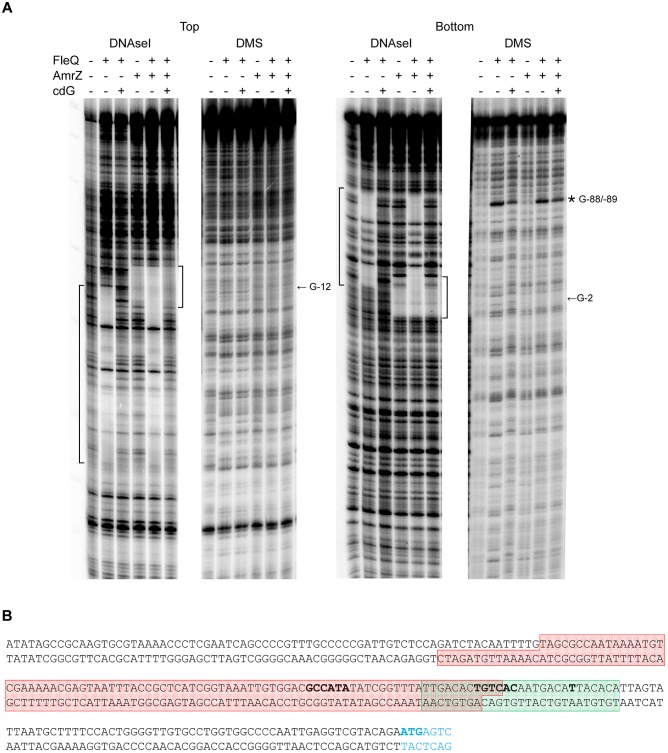
Identification of the FleQ binding site in the *wss* promoter region by footprinting analysis. **(A)** DNAse I and DMS footprint. DNA probes corresponding to the *wssA* upstream region 5′ end-labeled on either the top or the bottom strand were prepared and incubated without (lanes -) and with FleQ (1 μm) and/or AmrZ (50 nM) and c-di-GMP (0.5 mM). After partial digestion with DNase I or treatment with DMS and partial digestion with piperidine, the DNAs were subjected to urea-PAGE. Nucleotide sequences protected by FleQ and AmrZ are indicated on the left and right, respectively, of each panel; ^∗^, indicates hyperreactivity. **(B)** Localisation of the FleQ binding site at the *wss* promoter. The boxes indicate the regions protected from DNAse I by FleQ (red) and AmrZ (green) in the top and bottom strands. The -10 and -35 regions and the *wss* transcriptional start site are in bold.

DNase I and DMS footprinting analyses were performed to identify the FleQ binding site at the *wss* promoter region. FleQ protected a region of about 100 bp against DNase I: from -94 to -10 at the top strand and from -108 to -13 at the bottom strand (Figure 4) of the *wssA* upstream region. Therefore, the position of the FleQ binding site (overlapping the -10 and -35 regions of the *wss* promoter) is in agreement with the transcriptional repression of this promoter observed *in vivo* under low c-di-GMP levels. Surprisingly, we could not detect protection from DMS methylation in the presence of FleQ either in the top or bottom strand. We only observed a hyperreactivity at Gs -88 and -89 in the bottom strand (Figure 4). In the presence of c-di-AMP, GTP, and ATP FleQ remained bound to the DNA with a DNase I and DMS footprint similar to that observed with the protein alone (Supplementary Figure S4). However, in the presence of c-di-GMP, the protection against DNAse I conferred by FleQ was lost, suggesting that most of the protein released from this position of the DNA. Nonetheless, the DMS hyperreactivity observed in the presence of FleQ was obvious, suggesting that some protein was still bound although the FleQ interaction with the DNA had changed.

### Regulation of Cellulose Production by FleQ and AmrZ

We have shown that FleQ binds the wssA1-2 fragment and the presence of c-di-GMP, but not c-di-AMP, partially disrupts the FleQ-DNA complex (Figure 3C and Supplementary Figure S3). Adding AmrZ together with FleQ to the EMSA further retarded the mobility of the DNA, indicating that both proteins were bound to the wssA1-2 fragment at the same time. Furthermore, the addition of c-di-GMP to the AmrZ-FleQ-DNA specifically increased the appearance of the AmrZ-DNA complex, indicating that c-di-GMP does not influence AmrZ-DNA binding (Figure 3C). This was confirmed with DNase I and DMS footprinting assays performed in the presence of AmrZ. This regulator bound to the *wssA* promoter protecting the region from -20 to +8 against DNAse I, and the -12 G at the top strand and the -2 G at the bottom strand from DMS methylation, as was previously shown (Prada-Ramírez et al., 2016). The protection remained the same in the presence of FleQ, alone or together with c-di-GMP, c-di-AMP, ATP or GTP, suggesting that AmrZ is able to stay bound to the DNA even when FleQ dissociates (Figure 4). Accordingly, *in vitro* transcription was 50% reduced by AmrZ regardless of the presence of c-di-GMP and further diminished when both proteins were present in the reaction, whereas the presence of c-di-GMP partially recovered it (Figure 2).

In summary, the *in vitro* assays show that AmrZ and FleQ are able to simultaneously bind to the DNA. Thus, we constructed and characterized a double *amrZ/fleQ* mutant at transcriptional and cellulose production levels to find out whether AmrZ and FleQ were co-repressing *wss* expression. We analyzed *wssB* expression by qRT-PCR under different intracellular c-di-GMP levels and observed that the mRNA levels were maximum in the double *amrZ/fleQ* mutant (higher than in the wild type background), regardless of the presence of PleD^∗^ (Figure 1A). These results indicate that the deletion of AmrZ and FleQ has an additive effect inducing *wss* expression to higher levels than the ones observed in the *fleQ* or *amrZ* single mutants under physiological c-di-GMP conditions. Furthermore, in the absence of both AmrZ and FleQ, no differences in the *wssB* mRNA levels were observed when the c-di-GMP levels were raised. Indeed, for the *wss* transcription to be maximal independently of the c-di-GMP levels, it is sufficient to eliminate the repressor effect of both regulators (Figure 1A).

In terms of cellulose production, the amount of cellulose synthesized by the double mutant under physiological c-di-GMP conditions was higher than in the *fleQ* or *amrZ* single mutants, and also maximum regardless of the presence of PleD^∗^ (Figure 1B). However, in contrast to the *wss* transcription (Figure 1A), the amount of cellulose produced by the *amrZ/fleQ* mutant was not higher than that observed in the wild type in the presence of PleD^∗^ (Figure 1B), suggesting that cellulose detection seems to reach a maximum under the condition tested.

### Cross-Regulation of *fleQ* and *amrZ* and Influence of c-di-GMP

**FIGURE 5:**
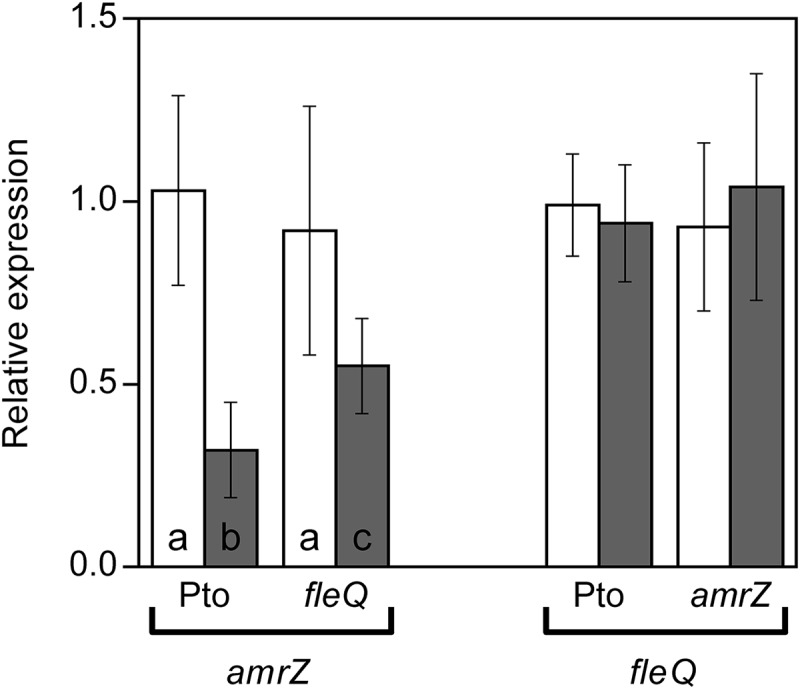
Expression of *amrZ* and *fleQ*. Total RNAs were obtained from bacteria grown in MMR at 20°C for 24 h. Results show qRT-PCR of *amrZ* in the wild type (Pto) and *fleQ* mutant and of *fleQ* in the wild type and *amrZ* mutant, with pJB3Tc19 (in the absence of *pleD^∗^*, white bars) or with pJB3pleD^∗^ (in the presence of *pleD*^∗^, gray bars). Expression values were normalized with the housekeeping gene *gyrA* and referred to the wild type condition in the absence of *pleD*^∗^. The graph shows the average mRNA levels and error bars correspond to the standard deviation of three biological replicates; a-c denote ANOVA categories with significant differences (*P* < 0.01).

FleQ and AmrZ share an important part of their regulons in *P. fluorescens* F113 (Blanco-Romero et al., 2018). Furthermore, FleQ transcriptionally represses *amrZ* expression and *fleQ* is under strong AmrZ repression both in *P. aeruginosa* (Tart et al., 2006) and *P. fluorescens* (Martínez-Granero et al., 2012). In order to determine the cross-regulation of these two transcriptional regulators in Pto DC3000, we measured the expression of *amrZ* in a *fleQ* mutant and *vice versa*, and compared them to the wild type by RT-qPCR (Figure 5). Under physiological c-di-GMP conditions, we observed no significant differences in the expression of both genes in any of the genetic backgrounds. Therefore, neither FleQ is controlling the expression of *amrZ* nor AmrZ regulates *fleQ*, at least under the conditions tested. Interestingly, *amrZ* expression, but not *fleQ*, was slightly reduced under high c-di-GMP, both in the presence and in the absence of FleQ, decreasing 3.3-fold in the wild type strain and 1.6-fold in the *fleQ* mutant (Figure 5).

### FleQ and AmrZ Interrelated Regulation of Other Pto DC3000 Traits

The results obtained so far indicate a remarkable regulatory cooperation between AmrZ and FleQ on cellulose production. But these two important transcriptional regulators are also involved in the regulation of other phenotypes in Pto DC3000 that are crucial for the interaction with the plant host (Nogales et al., 2015; Prada-Ramírez et al., 2016). In order to elucidate whether AmrZ and FleQ co-regulate other Pto DC3000 functions, we also studied the motility and virulence behavior of the *amrZ/fleQ* double mutant in comparison with its respective single mutants and the wild type.

#### Motility

**FIGURE 6:**
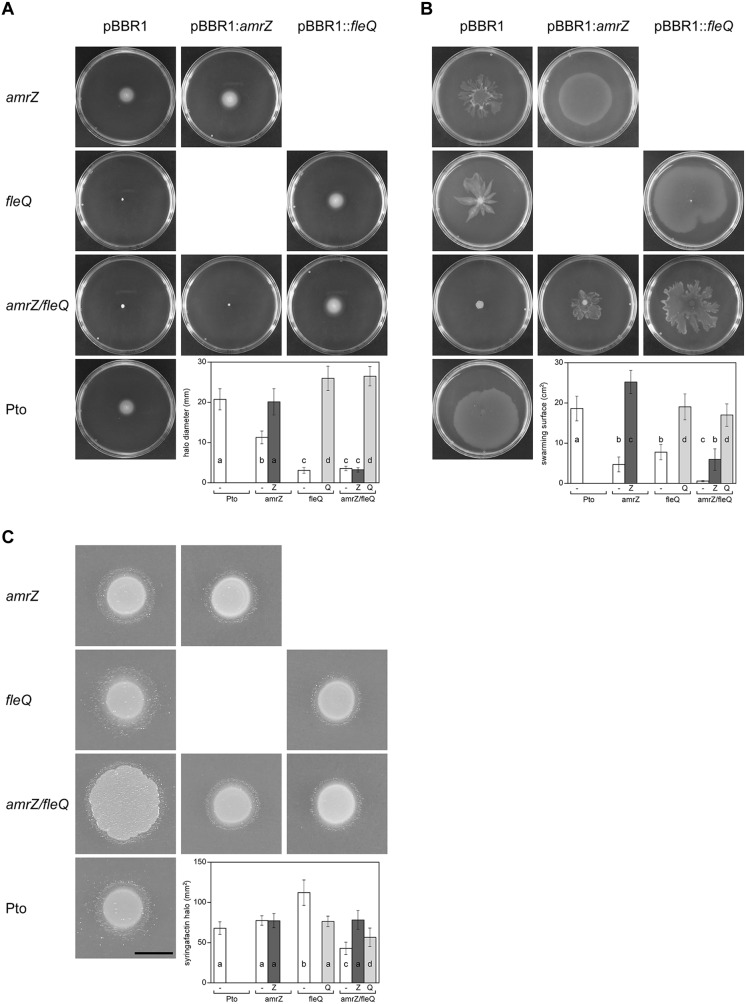
Motility of Pto DC3000 and its mutants. Observation and quantification of motility and syringafactin production were carried out in the different genetic backgrounds. Mean values are indicated for the different strains with the empty plasmid pBBR1MCS (white), pBBR1MCS::*amrZ* (dark gray), and pBBR1MCS::*fleQ* (light gray). Error bars correspond to the standard deviation of at least three biological replicates. a–d denote ANOVA categories with significant differences (*P* < 0.01). **(A)** Swimming assays. The indicated strains were punctured in the center of LB (0.3% agar) plates and incubated 48 h at 20°C, when pictures were taken and swimming halos measured. The graphic shows the respective average diameter of the halos. **(B)** Surface motility assays. Bacterial suspensions of the indicated strains were deposited on the surface of PG-agar (0.5% agar) plates and incubated 24 h at 20°C, when pictures were taken. The respective areas of motility were calculated with ImageJ. **(C)** Syringafactin production. Comparison of surfactant-induced halos around bacterial colonies grown on LB (1% agar) for 24 h at 20°C and visualized by the atomized oil assay. Bar = 1 cm. The graphic shows the respective biosurfactant areas.

FleQ is the master regulator of flagellar biogenesis and AmrZ also functions as a positive regulator of flagellar production in Pto DC3000 (Nogales et al., 2015; Prada-Ramírez et al., 2016). The *amrZ/fleQ* mutant exhibited no swimming motility, as the *fleQ* mutant, and only complementation with the *fleQ* gene restored the wild type phenotype (Figure 6A). These results suggest that swimming motility is not co-regulated by AmrZ and FleQ, the latter being the only one required. Interestingly, this is not the case for swarming, the other flagella-dependent movement in Pto DC3000 (Nogales et al., 2015). In contrast to the single mutants, the double mutant exhibited no swarming motility. Furthermore, complementation with the *fleQ* gene restored the *amrZ* phenotype, whereas complementation with the *amrZ* gene restored the *fleQ* phenotype (Figure 6B). Altogether, these results indicate that FleQ and AmrZ are co-regulating swarming motility, and that this regulation is exerted over factor(s) other than flagellar biogenesis.

We previously showed that FleQ negatively regulates syringafactin production in Pto DC3000 (Nogales et al., 2015), therefore we studied the behavior of the *amrZ* and the double mutant in this regard. We observed that the AmrZ single mutant produced similar levels of syringafactin to the wild type (Figure 6C), whereas the *fleQ* mutant produced significantly more (Nogales et al., 2015). However, the double mutant *amrZ/fleQ* although it spread more on the plates probably due to the increased production of cellulose, produces 45% less syringafactin than the wild type. Furthermore, the reintroduction of either of these two transcriptional regulators increased back the syringafactin levels (Figure 6C). Syringafactin is one of the biosurfactants produced by Pto DC3000 which is required for swarming motility (Berti et al., 2007; Nogales et al., 2015), likely being one of the factors that are also co-regulated by AmrZ and FleQ.

#### Virulence

**FIGURE 7:**
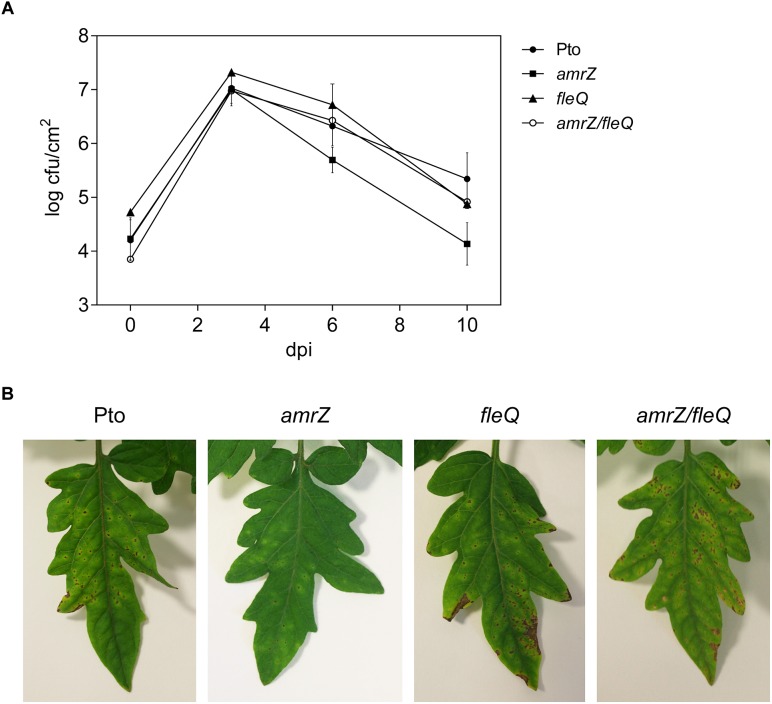
Bacterial growth and symptom development on tomato leaves. **(A)** Time course of growth of Pto DC3000 and the *amrZ*, *fleQ*, *amrZ/fleQ* mutants in the primary leaves of tomato plants. Colony forming units (cfu) were quantified at 0, 3, 6, and 10 days post-inoculation (dpi) with approximately 10^8^ cfu/ml by spray. Data represent the averages from three experiments with their standard deviations. **(B)** Development of symptoms induced on tomato leaves 10 days after inoculation with Pto DC3000 and different mutants.

To study the impact of the simultaneous lack of AmrZ and FleQ on Pto DC3000 virulence, we carried out infection experiments in tomato plants with the wild type and the *amrZ/fleQ* mutant and the single mutants as controls (Figure 7). We compared the abilities of the wild type strain with the *amrZ*, *fleQ* and *amrZ/fleQ* mutants to survive and multiply in tomato leaf tissues by monitoring bacterial populations and development of disease symptoms for 10 days after inoculation by spray. All mutants behaved, similarly, to the wild type in tomato leaves: their populations reached a maximum at 3 dpi (1.2 × 10^7^–2.1 × 10^7^ cfu/cm^2^), and then decreased to 1.9 × 10^4^–4.3 × 10^5^ cfu/cm^2^ at 10 dpi. As was seen before, the *amrZ* single mutant populations decayed faster than the other strains (Prada-Ramírez et al., 2016), and *fleQ* and the double mutant behavior was indistinguishable from the wild type (Figure 7A) (Nogales et al., 2015). The disease symptoms generated by the wild type and all the mutants assayed were also similar: small water-soaked lesions that became chlorotic 2–3 days after the inoculation and subsequently turned brown. However, the severity of the symptoms caused by the double mutant and *fleQ* was similar to the wild type, whereas the *amrZ* mutant provoked far less lesions (Figure 7B). Thus, the reduced virulence of the *amrZ* mutant was partially recovered by introducing a second mutation on *fleQ*, suggesting that these two transcriptional regulators behave as antagonists in the co-regulation of one or more phenotypes with influence on Pto DC3000 virulence.

## Discussion

EPSs, like other macromolecules, are energy-costly produced polymers and therefore their production is usually tightly regulated in bacteria (Schmid et al., 2015). Cyclic-di-GMP has been demonstrated to be a universal second messenger that controls the production of many such macromolecules as a part of a bacteria strategy for transitioning from an individual to a more associated way of life (biofilm), for enhancing their survival in the environment or for facilitating their interaction with eukaryotic hosts (Römling et al., 2013). Cellulose production is activated at its final step by c-di-GMP at the post-translational level *via* its direct binding to the glycosyltransferase involved in its polymerisation (Morgan et al., 2014). Furthermore, c-di-GMP also regulates cellulose production at the transcriptional level, activating the expression of the biosynthetic genes in different bacteria (Weber et al., 2006; Fazli et al., 2011; Barnhart et al., 2013; Hengge, 2016). This multilevel regulation of EPS production by c-di-GMP, at the transcriptional and post-translational levels, is a demonstration of the so-called sustained sensing mechanism. In bacteria, sustained sensing involves multiple receptors controlling different steps in the same biological process, which provides additional levels of regulation being physiologically meaningful (Orr et al., 2016).

In this study, we show that the regulation of cellulose production in Pto DC3000 is very complex since it takes place at several stages and most likely requires other unknown proteins that interact with c-di-GMP. The cellulose biosynthetic operon is co-regulated by at least two transcriptional regulators in this bacterium, FleQ and AmrZ. Under physiological c-di-GMP levels, both regulators have been shown to bind directly to the promoter region of the *wss* operon inhibiting its expression. The additive effect of the double *amrZ/fleQ* mutation on the expression of *wss* in that condition (Figure 1), together with the fact that they do not seem to regulate each other at the transcriptional level (Figure 5), suggest that FleQ and AmrZ are independent regulators converging in the control of the cellulose synthesis operon, unlike it has been described for other species of the genus (Blanco-Romero et al., 2018). Thus, in *P. fluorescens* and *P. aeruginosa*, both transcriptional regulators are part of the same regulatory cascade oriented toward the production of EPS (Tart et al., 2005; Martínez-Granero et al., 2012; Fazli et al., 2014). Furthermore, the described positive role of AmrZ over exopolysaccharide production in *P. fluorescens* F113 is then in stark contrast to the repressive function observed over *wss* operon in Pto DC3000. Finally, the co-regulation exerted by AmrZ and FleQ does not seem to be limited to cellulose production in Pto DC3000 and affects other phenotypes important for the interaction with the plant host, such as swarming motility, most likely through the regulation of syringafactin production (Figure 6), or symptom development (Figure 7). It is important to note that AmrZ and FleQ behave as antagonists in the co-regulation of several phenotypes influencing Pto DC3000 virulence.

In Pto DC3000 the behavior of these transcriptional regulators in cellulose production is completely different under high c-di-GMP levels. On one hand, AmrZ repress the transcription of the *wss* operon independently of c-di-GMP. However, although the *amrZ* mutation generates a discrete increase in transcription of the *wss* operon under physiological levels of c-di-GMP, this renders a strong production of cellulose (Figure 1). AmrZ not only repress the transcription of the *wss* operon, but also regulates different DGCs in Pto, like *morA* and *adcA/gcbA* (Prada-Ramírez et al., 2016), in a similar way than in *P. aeruginosa* or *P. fluorescens* F113 (Jones et al., 2014). Thus AmrZ may indirectly modulate the specific c-di-GMP pool involved in energizing the cellulose production in at least two different ways: (i) indirectly influencing the *wss* transcription *via* FleQ and (ii) at the post-translational level activating the synthase complex through the WssB PilZ domain. The complexity is even higher if we consider that *amrZ* mRNA levels are negatively affected by c-di-GMP (Figure 5).

FleQ, on the other hand, regulates Pto *wss* operon in a c-di-GMP dependent manner, converting from a repressor to an activator upon c-di-GMP binding. *In vitro* experiments indicated that FleQ binds the *wss* promoter in the absence of c-di-GMP. This is evidenced by a shift in EMSA and a protected region of about 100 bp (from -94 to -10 at the top strand and from -108 to -13 at the bottom strand) in the DNAse I footprint experiments. This binding is disrupted by c-di-GMP since the protection is lost and a DMS hyperreactivity at Gs -88 and -89 was observed in the bottom strand (Figure 3, 4). These results suggest that the FleQ interaction with the DNA is drastically changed upon c-di-GMP binding, differently of *P. aeruginosa*, where FleQ remains bound to the *cdrAB*, *pel*, *psl*, and PA2440 promoters in the presence of c-di-GMP (Baraquet et al., 2012; Baraquet and Harwood, 2016). The FleQ dual regulatory role is particularly evident *in vivo*, since the expression of the cellulose synthesis operon is intermediate and independent of the c-di-GMP levels in a *fleQ* mutant. It also produces less cellulose when intracellular levels are raised, losing the wrinkly colony phenotype of the wild type (Figure 1B). This dual behavior of FleQ depending on the c-di-GMP levels has been reported for other EPS, like *pel* and *psl*, and biofilm gene promoters in *P. aeruginosa* (Baraquet et al., 2012; Baraquet and Harwood, 2016). Therefore, it seems that FleQ-c-di-GMP-dependent regulation is conserved in the course of evolution among different *Pseudomonas*, controlling different EPSs depending on the species: e.g., Pel in *P. aeruginosa* (which is absent in Pto) and cellulose in Pto (which is absent in *P. aeruginosa*). In addition, it should be noticed that the positive effect of FleQ-c-di-GMP over cellulose transcription is remarkably relevant when AmrZ is present (Figure 1, 2). AmrZ and FleQ binding sites are close, indeed overlapping by few nucleotides around the -10 region. When both transcriptional regulators are bound to their respective regions under low c-di-GMP levels, they may directly interact. Thus, even when FleQ and/or c-di-GMP do not seem to have any effect on the AmrZ binding, it is plausible that the conformational changes induced by FleQ-c-di-GMP in the DNA may reduce the role of AmrZ as a repressor of the *wss* promoter. Future experiments will be carried out to shed light over the possible interaction of FleQ-c-di-GMP and AmrZ and with other proteins, like FleN. FleN is an ATPase which has been described as a FleQ antagonist in regulating the flagellar genes in *P. aeruginosa*, but has been also demonstrated to act as a synergistic factor of FleQ in controlling the two biofilm matrix coding operons in *P. putida* KT2440 (Baraquet and Harwood, 2013; Nie et al., 2017).

Overall, the results obtained in this work indicate that the regulation of the cellulose production in Pto DC3000 is another example of sustained sensing (Orr et al., 2016) by using c-di-GMP as a signaling molecule to control multiple steps: the expression of the biosynthetic machinery and subsequently its activation, assuring a rapid but precise response to environmental cues, including those required for the interaction with the plant host. In this sense, cellulose seems to have a diverse role in the bacterial life cycle depending on the model studied (Matthysse, 1983; Prigent-Combaret et al., 2012; Prada-Ramírez et al., 2016; Castiblanco and Sundin, 2018). Therefore, it is necessary to study the still unknown elements in Pto DC3000, such as the environmental cues triggering cellulose production, the sensors and signaling cascades that lead to the expression of AmrZ, FleQ and the DGC(s) or PDE(s) involved.

## Materials and Methods

### Bacterial Strains, Plasmids, and Growth Conditions

The bacterial strains used in this study are listed in Table 1. *E. coli* and *P. syringae* pv. tomato DC3000 strains were routinely grown in Luria-Bertani (LB) medium (Sambrook et al., 1989) at 28°C. Pto DC3000 was also grown in MMR (7 mM Na-glutamate, 55 mM mannitol, 1.31 mM K_2_HPO_4_, 2.2 mM KH_2_PO_4_, 0.61 mM MgSO_4_, 0.34 mM CaCl_2_, 0.022 mM FeCl_3_, 0.85 mM NaCl) minimal medium (Robertsen et al., 1981) at 20°C. When required, other compounds such as antibiotics were added: ampicillin (100 μg/ml), chloramphenicol (30 μg/ml), gentamicin (2–10 μg/ml), kanamycin (25 μg/ml), rifampicin (10 μg/ml), and tetracycline (10 μg/ml).

**Table 1:** Bacterial strains and plasmids used.

Strain or Plasmid	Relevant characteristics	References
**Strains**		
*P. syringae* pv. tomato		
DC3000	Wild type, Rif^R^	Cuppels, 1986
*fleQ*	*fleQ*::ΩKm; Rif^R^ Km^R^	Vargas et al., 2013
*amrZ*	*amrZ*::Gm; Rif^R^ Gm^R^	Prada-Ramírez et al., 2016
*amrZ/fleQ*	*amrZ*::Gm *fleQ*::ΩKm; Rif^R^ Gm^R^ Km^R^	This work
*E. coli*		
One Shot BL21star (DE3)	F^-^ *ompT hsdS_B_* (r_B_^-^, m_B_^-^) *gal dcm rne131* (DE3)	Thermo Fisher Scientific
**Plasmids**		
pBBR1-MCS	Cm^R^; cloning vector	Kovach et al., 1995
pBBR1-MCS::*amrZ*	Cm^R^; pBBR1-MCS with a 628 pb fragment containing the *amrZ* gene flanked by the BamHI and XbaI restriction sites	This work
pBBR1-MCS::*fleQ*	Cm^R^; pBBR1-MCS with a 2001 pb fragment containing the *fleQ* gene flanked by the XhoI and BamHI restriction sites	This work
pBBR1-MCS2::*amrZ*	Km^R^; pBBR1-MSC2 with a 629 pb fragment containing the *amrZ* gene flanked by the BamHI and XbaI restriction sites	Prada-Ramírez et al., 2016
pBluescript::*amrZ*-Gm	Ap^R^ Gm^R^; pBluescript derivative containing a 2.207 pb PstI/HindIII fragment with the *amrZ* ORF and flanking regions from Pto DC3000 and the gentamycin cassette from p34S-Gm cloned into the SphI site of *amrZ*	Prada-Ramírez et al., 2016
pET29a(+)	Km^R^; protein expression vector, T7 promoter	Novagen
pAMRZ-Pto	Km^R^; pET29a(+) bearing a 336 bp NdeI/XhoI fragment containing *amrZ*	Prada-Ramírez et al., 2016
pET29Q	Km^R^; pET29a(+) bearing a 1481 bp NdeI/XhoI fragment containing *fleQ*	This work
pJB3Tc19	Ap^R^ Tc^R^; cloning vector, P*_lac_* promoter	Blatny et al., 1997
pJB3pleD^∗^	Ap^R^ Tc^R^; pJB3Tc19 bearing a 1423 bp XbaI/EcoRI fragment containing *pleD^∗^*	Pérez-Mendoza et al., 2014


*Ap^*R*^, Cm^*R*^, Gm^*R*^, Km^*R*^, Rif^*R*^, Tc^*R*^ stand for resistance to ampicillin, chloramphenicol, gentamicin, kanamycin, and rifampicin, respectively.*

EPS production can be detected and even quantified using dyes such as CF or CR. CR binds to neutral or basic polysaccharides and some proteins, whereas CF is more specific and binds to β(1-4) and β(1-3) glycosidic bonds, like those present in cellulose, and positive colonies fluoresce under UV light (Spiers et al., 2002). Colony morphology and EPS production were visualized on MMR plates with CR (50 μg/ml) and CF (50 μg/ml).

Plasmid pJBpleD^∗^ (Pérez-Mendoza et al., 2014) was constructed by subcloning the XbaI/EcoRI fragment containing the *pleD^∗^* gene, which carries four point mutations and exhibit constitutive diguanylate cyclase activity from pRP89 plasmid (Paul et al., 2004) into the broad host range vector pJB3Tc19 (Blatny et al., 1997) previously digested with the same restriction enzymes. This constitutive expression of *pleD^∗^* in Pto DC3000 stably increases the intracellular c-di-GMP levels independently of its regulation by phosphorylation (Pérez-Mendoza et al., 2014).

### Plasmid and Strain Construction

We constructed plasmids bearing *amrZ* and *fleQ* genes for *in trans* complementation of the respective deficient mutants. For *amrZ*, we digested the pBBR1-MCS2::*amrZ* plasmid (Prada-Ramírez et al., 2016) with BamHI and XbaI and the fragment was ligated to pBBR1-MCS (Kovach et al., 1995) digested with the same enzymes. For *fleQ*, we digested pBBR1-MCS5::*fleQ* (Nogales et al., 2015) with XhoI and BamHI and the fragment was ligated to pBBR1-MCS (Kovach et al., 1995).

We also constructed the pET29Q plasmid for native FleQ overexpression and purification. The *fleQ* gene was cloned into pET29a(+) as a NdeI-XhoI fragment after PCR of chromosomal DNA with the oligonucleotides fleQ-NdeI (aaaaacatATGTGGCGTGAAATCAAG) and fleQ-XhoI (aaa actcgagtcaTCAATCCTCCGCCTGTTC).

The *amrZ/fleQ* double mutant was obtained by electroporating the Ap^R^Gm^R^ suicide plasmid pBluescript::*amrZ*-Gm into the *fleQ* mutant (Prada-Ramírez et al., 2016). Transformants that acquired the inactivated gene were selected (Gm^R^) and, among them, we screened for the Ap (300 μg/ml)-sensitive ones, indicative to be the result of a double-recombination event. One of the Gm^R^ Km^R^/Ap^S^ clones was chosen and confirmed by Southern blot to have the wild type *amrZ* gene replaced by the mutant allele *amrZ*-Gm (not shown).

Plasmid transformation of Pto DC3000 strains were carried out by electroporation. Electro-competent cells were prepared according to Choi et al. (2006), mixed with DNA (0.3–0.5 μg of DNA per ml of cell suspension) in 0.1 cm cuvettes and electroporated with a high-voltage pulse (1.800 V) for 5 ms by using an Eppendorf electroporator 2510. Transformants were selected in LB agar plates supplemented with the appropriate antibiotics.

### Motility Experiments

For swimming assays, the different strains were grown on LB plates for 48 h, resuspended in 10 mM MgCl_2_ and adjusted to an OD_660_ of 2.0. 2 μl aliquots were stabbed into LB plates (0.3% agar) and incubated 48 h at 20°C. The swimming halo diameters were measured after 48 h. For swarming motility assays, the 2 μl aliquots were dropped in the center of PG-agar plates (0.5% protease peptone No. 3 (Difco 212693) and 0.2% glucose with 0.5% Difco Bacto-Agar) and incubated at 20°C and observed after 24 h. Three replicas were used for each strain in different plates, and the experiment was repeated with three independent cultures (a total of 9 motility plates per strain).

### Syringafactin Production

Syringafactin was detected with the atomized oil assay previously described (Nogales et al., 2015). Pto DC3000 and mutants were grown on LB plates for 48 h and resuspended in sterile milliQ water. 10 μl aliquots (OD_660_ = 1.0) were pipetted onto the surface of LB plates, incubated 24 h at 20°C and then sprayed with a mist of mineral oil. The diameter of the visible halo of brighter oil drops was measured and the area of the producing bacterial colony was estimated and subtracted from that of the surfactant halo to yield the adjusted halo area. Three plates were used for each strain, and the experiment was repeated with four independent cultures.

### Quantification of Cellulose Production

Calcofluor binding assays by the different strains were performed as follows: bacteria were suspended from fresh LB plates in 10 mM MgCl_2_, diluted into 10 ml flasks containing MMR supplemented with CF (100 μg ml^-1^ final concentration) to an initial OD_660_ of 0.05, and incubated at 20°C under agitation for 24 h. Cultures were then centrifuged for 10 min at 4000 rpm, supernatant containing unbound CF broth was removed and the pellet was then suspended in 10 ml of distilled water. CF binding measurements for six biological replicates of each strain were performed in a PTI fluorimeter (Photon Technology International), after confirming a similar growth of all strains, and expressing the results in arbitrary units ± standard deviation.

### RNA Preparation and Assays

Pto DC3000 and mutant strains were grown in MMR and incubated at 20°C for 24 h. The cells were harvested, pelleted, frozen in liquid nitrogen and processed for RNA isolation using TRI ReagentLS (Molecular Research Center, Cincinnati, OH, United States) as described before (Vargas et al., 2011).

For RT-qPCR, total RNA (1 μg) treated with Rnase-free Dnase I Set (Roche) was reversely transcribed using Superscript II reverse transcriptase (Invitrogen) and random hexamers (Roche) as primers. Quantitative real-time PCR was performed on an iCycler iQ5 (Bio-Rad, Hercules, CA, United States). Control PCR reactions of the RNA samples not treated with reverse transcriptase were also carried out to confirm the absence of contaminating genomic DNA. The specific primer pairs used to amplify cDNA are listed in Table 2; primer efficiency was optimal for all the pairs (∼100%). Real-time RT-PCRs were performed in triplicate from three biological replicates and relative transcript abundance was calculated by the ΔΔCt method normalizing to the housekeeping gene *gyrA* (Vargas et al., 2011). Results shown are the means and standard deviations of at least three independent experiments with three replicates.

**Table 2:** Oligonucleotides used in this work.

Name	Sequence (5′ → 3′)	Characteristics	Fragment
**Oligonucleotides used to generate the EMSA probes**			
fleSF	ACCACCCTGGTCGAGAAG	Amplify a 314 bp fragment upstream *fleS* CDS, between nucleotides 2141382 and 2141695 of the Pto DC3000 genome	fleS1
fleSR	CTGTGCGGACATCTGATTG		
fleS3-F	CGACATGCCACTACCGAT	Amplify a 327 bp fragment upstream *fleS* CDS, between nucleotides 2140613 and 2140939.	fleS3
fleS3-R	GCGTGACGACATAGCGAC		
EcoRI_PhrpL	aagaattcAGTTGATAAAGCTCACCGAG	Amplify a 481 bp fragment upstream *hrpL* CDS, between nucleotides 1542353 and 1542819.	PhrpL
PstI_PhrpL	aactgcaGGTGTACAAGCCCTATAGC		
wssAF	CCAGCCACTGATTTAATTCG	Amplify a 309 bp fragment upstream *wssA* CDS, between nucleotides 1119694 and 1119985.	wssA1
wssAR	CGTCAGCACGACTCATTC		
wssAF2	CGCCAATAAAATGTCGAA	Amplify a 323 bp fragment upstream *wssA* CDS, between nucleotides 1119829 and 1120134.	wssA2
wssAR2	TGGTTGCTCGATAGACGG		
wssAF	CCAGCCACTGATTTAATTCG	Amplify a 323 bp fragment upstream *wssA* CDS, between nucleotides 1119694 and 1120134.	wssA1-2
wssAR2	TGGTTGCTCGATAGACGG		
wssG-F	GTTCGATTAATCCAGTGA	Amplify a 299 bp fragment upstream *wssH* CDS, between nucleotides 1131888 and 1132186.	wssH1
wssG-R	CCAACTGCCGATCAACAGAAC		
**Oligonucleotides used for RT-qPCR**			
amrZ+	GAAATCATTGCCCGCCTCGAACAAAGC		
amrZ-	TGGGCGATCAGCGAAACGAGAGCAT		
fleQ+	GCGAGAGGTTCTGTGCGTGCTGGTC		
fleQ-	GCAGCTCGACGGAAGAGTTTTCGCTCA		
gyrA+	GGCAAGGTCACCCGCTTCAAGGAAT		
gyrA-	GACCGCCACGCTTGTACTCAGGGAAC		
wssB+	GGTGTTCAACGCTGTGACGCAGGA		
wssB-	TGGCGCAGTGAAAGATCATCGAAACG		


### Protein Purification

AmrZ was purified as described before (Prada-Ramírez et al., 2016). For FleQ purification, the One Shot BL21star (DE3) (pET29a(+)::*fleQ*) cells were grown at 28°C in 2-L Erlenmeyer flasks containing 1 L of 2 × YT culture medium (Sambrook et al., 1989) supplemented with kanamycin (50 μg/ml). Protein expression was induced at an OD_660_ of 0.2–0.3 by adding 0.5 mM isopropyl β-D-1-thiogalactopyranoside and cultures were grown for another 20 h at 15°C, when they were harvested by centrifugation at 5000× *g*. The pellet resulting from a 500 ml culture was resuspended in 30 ml of buffer A (20 mM Tris-HCl pH 8.5, 300 mM NaCl, 0.1 mM EDTA) with protease inhibitor mixture (Complete^TM^, Roche) and broken by treatment with 20 μg/ml of lysozyme and French press. Following centrifugation at 13,000× *g* for 60 min, the FleQ protein was predominantly present in the soluble fraction. The supernatant was loaded onto a Hitrap Heparin HP column (5 ml, GE Healthcare) equilibrated with buffer A and eluted with a gradient of 0.4–1.5 M NaCl. Fractions containing FleQ were pooled and dialyzed against buffer C (20 mM Tris-HCl pH 8.5, 500 mM NaCl, 0.1 mM EDTA, 10% glycerol) for protein storage at -70°C. Protein concentrations were determined using the Bio-Rad Protein Assay kit.

### Fluorescence-Based Thermal Shift Assays

Fluorescence-based thermal shift (FTS) assays were performed using a BioRad MyIQ2 Real-Time PCR instrument. Each 25 μl assay mixture contained 1 μM FleQ and SYPRO Orange at 5× concentration in STAD [25 mM Tris-acetate pH 8.0, 8 mM Mg-acetate, 10 mM KCl, 3.5% (w/v) polyethylene glycol-8000 and 1 mM DTT]. The nucleotides were prepared as 10× stocks and aliquots of 2.5 μl were added to each well. Samples were heat denatured from 20 to 90°C at a ramp rate of 0.5°C/min. The protein unfolding curves, both in the absence and in the presence of the nucleotides, were monitored by detecting changes in SYPRO Orange fluorescence and the first derivative values (-dF/dt) from the raw fluorescence data were used to determine the melting temperature (Tm). All experiments were performed in triplicate.

### Electrophoretic Mobility Shift Assays (EMSA)

Different fragments (309, 323, and 458 bp) containing the *wssA* promoter region obtained from DC3000 chromosomal DNA by PCR were used as DNA probes (Table 2). The PCR product was isolated from an agarose gel by using the Nucleospin gel and PCR clean-up (Macherey-Nagel) and was radiolabeled at its 5′-ends with [γ-^32^P]ATP and T4 polynucleotide kinase. The labeled probe (20 nM) was then incubated with the indicated concentrations of purified FleQ and/or AmrZ in 10 μl of STAD [25 mM Tris-acetate pH 8.0, 8 mM Mg-acetate, 10 mM KCl, 3.5% (w/v) polyethylene glycol-8000 and 1 mM DTT] supplemented with 10 μg ml^-1^ of poly(dI-dC), and 200 μg ml^-1^ of bovine serum albumin. The reaction mixtures were incubated for 30 min at 4°C, and samples were run on 4% (w/v) native polyacrylamide gels (Bio-Rad Mini-Protean II) for 2 h at 50 V at room temperature in Tris-glycine (25 mM Tris, 200 mM glycine). The results were analyzed with Personal FX equipment and Quantity One software (Bio-Rad).

### DNA Footprints

The DNA probe was the 458-bp PCR fragment containing the *wssA* promoter region. For the footprint on the top strand, the PCR was carried out with primers wssA-F (end labeled with [γ-^32^P]ATP as described above) and wssA2-R. For the footprint on the bottom strand, the same primers were used, but in this case, wssA2-R was end-labeled. Purified labeled probe (20 nM) was incubated without or with FleQ (1 μM) and AmrZ (50 nM) in 50 μl reaction volume of STAD [25 mM Tris-acetate pH 8.0, 8 mM Mg-acetate, 10 mM KCl, 3.5% (w/v) polyethylene glycol-8000 and 1 mM DTT] supplemented with 10 μg ml^-1^ of poly(dI-dC), and 200 μg ml^-1^ of bovine serum albumin. Reaction mixtures were incubated for 30 min at 4°C before being treated with DNase I or DMS, as described previously (Rojas et al., 2003; Guazzaroni et al., 2004). The results were analyzed with Personal FX equipment and Quantity One software (Bio-Rad).

### *In vitro* Transcription Assays

Reactions (10 μl) were performed in STAD buffer with 0.5 μM FleQ, 50 nM AmrZ, 0.5 mM nucleotides, 4 Units of RNAse inhibitor (Roche) and 5 nM DNA template (wssA1-2 PCR fragment). After 30 min incubation at 4°C, 0.5 U σ^70^-holoenzyme (New England Biolabs) were added and the reactions were incubated for 5 min at 30°C before the addition of 1.2 μl of the following elongation mixture: 0.1 mM each for ATP, CTP and GTP, 0.05 mM UTP and 50 μCi [α-^32^P]UTP. After a further 15 min incubation at 30°C, the reactions were stopped by adding 3.7 μl of formamide sequencing dye. Samples were electrophoresed in a 6.5% (wt/vol) polyacrylamide denaturing sequencing gel. The results were analyzed with Personal FX equipment and Quantity One software (Bio-Rad).

### Statistical Analysis

Statistical comparison among different strains or conditions was performed by one-way ANOVA with *post hoc* Tukey HSD test using R.

## Author Contributions

MG designed the research. DP-M contributed to the conception and design of the work. AF, MG, MF, and DP-M performed the research. AF, MF, DP-M, JS, and MG analyzed the data. DP-M, MF, AF, JS, and MG wrote the manuscript. All the authors contributed to manuscript revision, read and approved the submitted version.

## Conflict of Interest Statement

The authors declare that the research was conducted in the absence of any commercial or financial relationships that could be construed as a potential conflict of interest.
