# Supplementary material for: AmrZ and FleQ Co-regulate Cellulose Production in Pseudomonas syringae pv. Tomato DC3000
Source: Front Microbiol. 2019 Apr 17;10:746. doi: 10.3389/fmicb.2019.00746 (PMC6478803; doi:10.3389/fmicb.2019.00746)

SUPPLEMENTARY FIGURES

Figure S1. Expression of the *wss* operon depending on the intracellular c-di-GMP levels.

A primer extension assay was carried out with total RNA from Pto DC3000 (pJB3Tc19) or Pto DC3000 (pJB3pleD*) grown in MMR to an OD_{660} = 0.5. 20 µg were used in reverse transcription reactions to generate the corresponding cDNA (lanes 1 and 2, respectively). Lane 3 contains the products of a sequencing reaction with DMS (bands correspond to G) carried on a PCR fragment marked with the same primer used in reverse transcription. The arrow points to the cDNA product obtained.

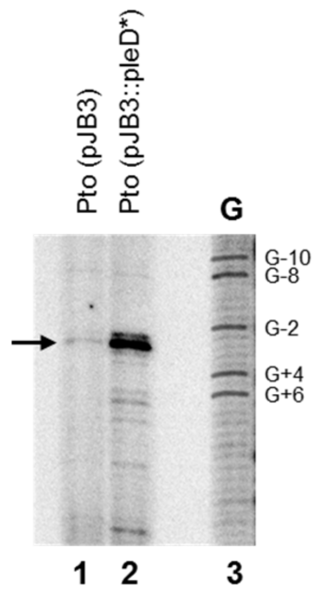

Figure S2. Fluorescence-based thermal shift assays of Pto DC3000 FleQ with different nucleotides.

Each point indicates the ΔTm, calculated as the Tm in the presence of ligand minus the Tm in the absence of ligand. c-di-GMP stabilizes the protein in a concentration dependent manner. In contrast, increasing concentrations of c-di-AMP does not have a stabilizing effect. Data are the means and standard deviations from three experiments.

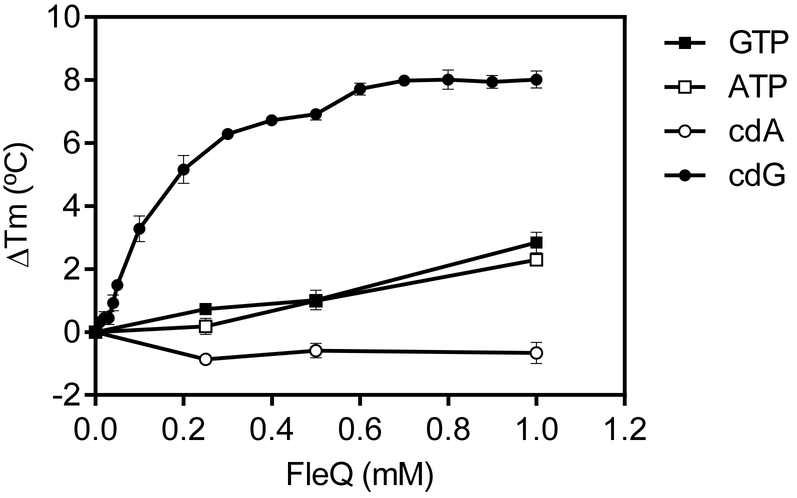

Figure S3. *In vitro* binding of FleQ to the *wss* promoter region in the presence of competitor DNA. Binding reactions were carried out as described in Experimental Procedures with 1 µM FleQ, when indicated, in the absence (-) and in the presence of c-di-GMP and c-di-AMP (0.5 mM) and cold DNA.

A. Competition with specific DNA. The cold DNA (wssA1-2 fragment) was added equimolar (1) and 5 times more concentrated (5) than the labelled DNA.

B. Competition with non-specific DNA. The cold DNA (PhrpL fragment) was added equimolar (1) and 5 times more concentrated (5) than the labelled DNA.

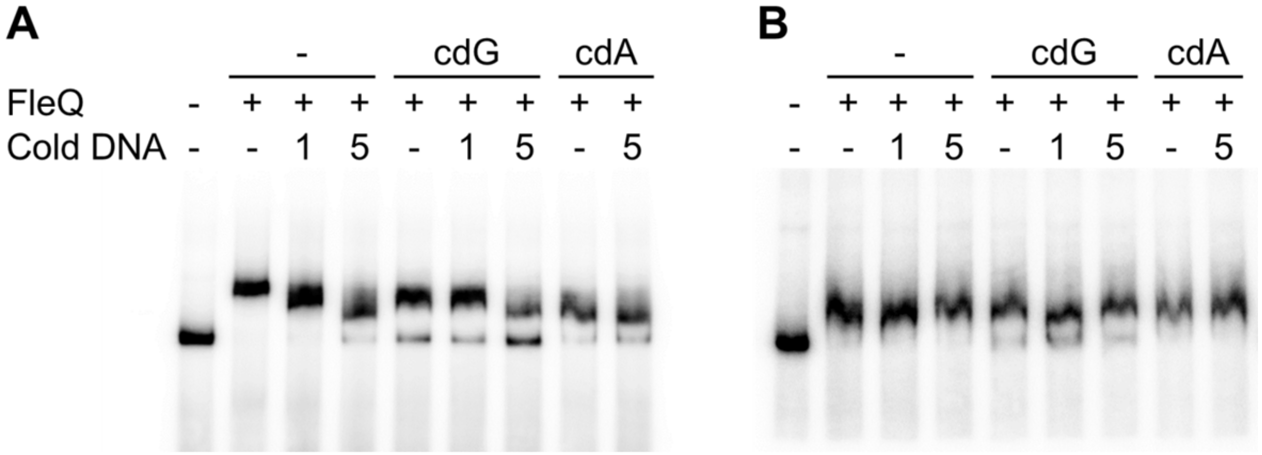

Figure S4. DNAse I and DMS footprint of FleQ at the *wss* promoter in the presence of different nucleotides.

DNA probes corresponding to the *wssA* upstream region 5' end-labelled on the bottom strand were prepared and incubated without (lanes -) and with FleQ (1 µm) and c-di-GMP (0.5 mM). After partial digestion with DNase I or treatment with DMS and partial digestion with piperidine, the DNAs were subjected to urea-PAGE. Nucleotide sequences protected by FleQ are indicated on the left and right, respectively, of each panel; *, indicates hyperreactivity.

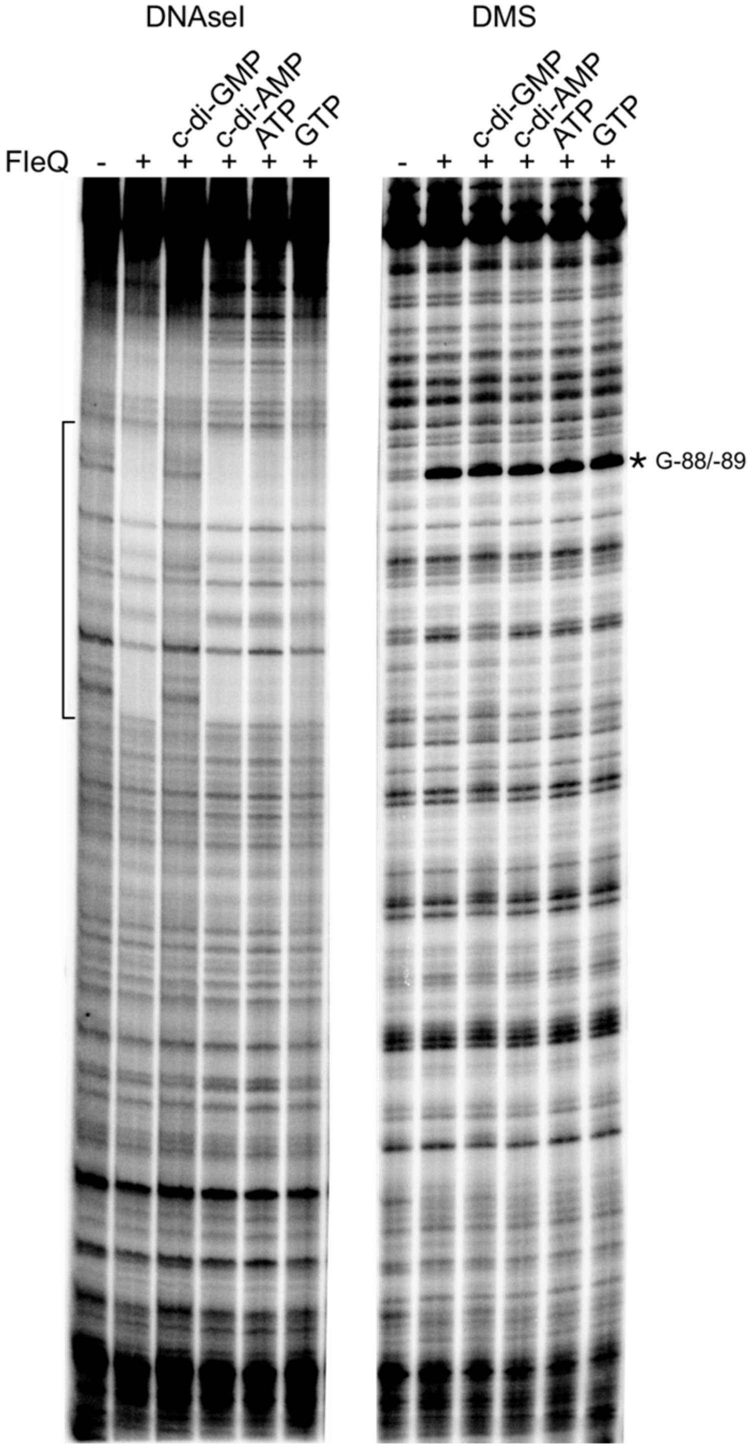

Supplement: Supplementary file 1 [file Presentation_1.pdf]
